# Data-Driven Models for Capacity Allocation of Inpatient Beds in a Chinese Public Hospital

**DOI:** 10.1155/2020/8740457

**Published:** 2020-01-07

**Authors:** Ting Zhu, Peng Liao, Li Luo, Heng-Qing Ye

**Affiliations:** ^1^West China Biomedical Big Data Center, West China Hospital/West China School of Medicine, Sichuan University, Chengdu 610064, China; ^2^Tencent.com, Shenzhen 518054, China; ^3^Department of Industrial Engineering and Engineering Management, Business School, Sichuan University, Chengdu 610064, China; ^4^Faculty of Business School, Hong Kong Polytechnic University, Hong Kong, China

## Abstract

Hospital beds are a critical but limited resource shared between distinct classes of elective patients. Urgent elective patients are more sensitive to delays and should be treated immediately, whereas regular patients can wait for an extended time. Public hospitals in countries like China need to maximize their revenue and at the same time equitably allocate their limited bed capacity between distinct patient classes. Consequently, hospital bed managers are under great pressure to optimally allocate the available bed capacity to all classes of patients, particularly considering random patient arrivals and the length of patient stay. To address the difficulties, we propose data-driven stochastic optimization models that can directly utilize historical observations and feature data of capacity and demand. First, we propose a single-period model assuming known capacity; since it recovers and improves the current decision-making process, it may be deployed immediately. We develop a nonparametric kernel optimization method and demonstrate that an optimal allocation can be effectively obtained with one year's data. Next, we consider the dynamic transition of system state and extend the study to a multiperiod model that allows random capacity; this further brings in substantial improvement. Sensitivity analysis also offers interesting managerial insights. For example, it is optimal to allocate more beds to urgent patients on Mondays and Thursdays than on other weekdays; this is in sharp contrast to the current myopic practice.

## 1. Introduction

Achieving optimal capacity allocation among multiple streams of demand is one of the main challenges faced by healthcare resource managers. The challenge concerns two aspects: (1) how much capacity quota should be allocated to each demand stream and (2) how to prioritize multiple streams of demand with distinct sensitivity to delays and distinct per-unit revenue, with the objective of simultaneously maximizing hospital revenue and equity. It is known that revenue and equity are two critical concerns when policy makers and hospital managers make capacity allocation decisions [1]. The allocation of hospital beds is a complex process and directly affects a hospital's operation effectiveness and patient experience. For example, patient leaving waiting queues to seek care services from other hospitals, emergency department (ED) being overcrowded, significant excess cost, and hospital bearing increase in patient mortality [2]. Uncertainties and random fluctuations inherent in both bed capacity and new demand substantially contribute to the difficulty in optimal decision-making in capacity allocation.

Multiple streams of hospitalization demand result in multiclass priority queues where patients are heterogeneous in their tolerance thresholds on waiting time and clinical situation-based service time. According to our field observations and the consultation with the nurses and physicians, an urgent patient with severe vital signs (such as the kidney cancer) who needs to be admitted to a hospital immediately may leave the waiting queue to seek service from other hospitals. In the worst case, this patient may pass away at home without being admitted. According to the health policy literature [3, 4], equity of access and responsiveness equity could be considered from a hospital's operations management perspective. In terms of equity, waiting time is one of the most important indicators [5, 6]. The unit waiting cost caused by urgent patients could be higher than regular patients. Revenue is generated by accepting patients, which varies by length of stay of patients with the same type of disease and insurance coverage. In contrast, regular patients (such as those with renal cyst) may not be so sensitive to delays, and these patients may require only simple treatments associated with a very short length of stay that generates a high unit revenue and is therefore beneficial to physicians' financial gains. Admitting too many regular patients could yield high hospital revenue, but also with increasing waiting cost derived from urgent patients, while admitting too many urgent patients could result in low hospital revenue, and also low waiting cost from regular patients. On the basis of the National Healthcare System, public hospitals in China must pursue equity and justice [1, 7, 8]. Moreover, except for the predetermined amount of social insurance, which is far below the medical expense, public hospitals are self-financing institutions in China's market economy system and have profit incentives [9]. Hence, maximizing both hospital revenue and equity is important and challenging for bed managers by balancing the trade-off between them.

There has been a great imbalance between hospital bed capacity and hospitalization demand in China, especially among large public hospitals. Hospital beds are critical resources but also limited because of the strict control of the expansion of public hospitals by the government health administration departments. The National Bureau of Statistics of China (2017) reported that the average number of inpatient beds for every 1,000 people is 5.72. Our collaboration, the West China Hospital (WCH) affiliated to Sichuan University, operates a large inpatient department with a capacity of over 4300 licensed beds shared by 44 specialty care units. The limited supply of inpatient beds has led to overcrowding with 6,000 elective patients waiting in the queue for admission every day. Every day the bed manager of WCH faces the challenge of deciding the number of backlogged urgent and regular elective patients to be admitted before accurate information about available bed capacity and new demand is realized or captured, in order to achieve bed management efficiency and effectiveness. The current allocation practice at WCH is a static fixed quota allocation policy myopically determined by the bed manager. Every day the bed scheduler allocates a fixed quota (70% to 80% with the exact value depending on the specific specialty care unit) of expected bed capacity to regular patients and the remaining to highly uncertain urgent patients, without considering the day of week patterns of patient arrivals, operating room schedules, and patient discharges. These patterns result in the daily fluctuations of demand and capacity, which should be considered in the allocation policy.

This paper studies a data-driven bed capacity allocation problem in a setting that both historical capacity and demand observations, as well as covariates (equivalently factors, features, and exogenous variables) associated with capacity and demand, are accessible, whereas their true distributions are unknown. The decision of each day is the bed quantity to be assigned to each demand stream: urgent elective patients and regular elective patients, with the objective of maximizing both revenue and equity. The bed capacity allocation in the practice of WCH is a static single-period stochastic optimization problem, and the newsvendor model is often used to aid decision-making in such type of problems. We establish a single-period newsvendor model, and data-driven methods are proposed to capture the performance of different optimization policies. Classical newsvendor problems consider a random variable, namely, demand, so the trade-off is to reduce the difference between the order quantity and the demand [10]. Here, the trade-off becomes the differences between the allocated bed quantities for urgent and regular elective patients and available capacity for them. Instead of assuming the probability distributions of the random variables, decision-makers will make use of past data and information to obtain direct predictions of the random variables.

Under such settings, there have been many works on obtaining the distributions of patient admission and departure as a function of decision variable or directly relaxing the assumption of distributions by using data-driven approaches. More interesting cases show that robust optimization methods [11–14] and nonparametric (“data-driven”) approaches [13, 15–18] are both effective alternatives for deriving the sample size bound and offering theoretical insights into the newsvendor problem. Feature-based data-driven approaches have been novelly developed and applied in the computer science realm (such as face recognition [19], medical imaging analysis [20], and machine learning [21]). In operations management and health care application, Ban and Rudin [18] considered a feature-based data-driven newsvendor problem and proposed two linear programming algorithms to find the optimal order quantity. They applied these algorithms to a nurse staffing problem and found that the expected cost of machine learning is lower than that of the benchmarking models. To the best of our knowledge, this research considers feature-based data-driven newsvendor problem under only demand uncertainty with one-dimension feature vector. However, we focus on how the proposed newsvendor model learns from in-sample data and offers a bed allocation quota with out-of-sample performance guaranteed.

In terms of applications, our work is associated with healthcare capacity allocation among multiple demand streams. As stated by Barz and Rajaram [22], hospital resource capacity cannot be stored for future time periods, and arrival patients are customers demanding a certain combination of resources and occupying the resources for a certain length of time at a price. In this case, a decision-maker faces two issues: (1) whether to satisfy a demand with a revenue or reject it at a cost when assuming only one patient will arrive and be served in a single period, with limited perishable resource being accessed by multipriority patients and (2) the amount of capacity to be reserved for high-priority demand in order to maximize both revenue and equity. Starting with Young [23], various models have been proposed in this realm, such as queueing models, stochastic optimization models, and simulation models. Gerchak et al. [24] proposed a stochastic dynamic programming model for determining the optimal reservation for emergency surgery patients under independent arrivals and unit capacity consumption, in which total capacity is known with certainty. Ayvaz and Huh [25] and Huh et al. [26] then extended the model to independent nonstationary arrivals and multiresource settings. In [25, 26], the authors also consider fixed capacity when capacity reservation decision is made. One reason for the fixed capacity may be that the application settings are in the operating rooms, where the total open time of a given operating room is a constant, while in our setting, bed capacity available for serving hospitalization in each period can be random. Considering the dynamic transition of system state, we extend the single-period model to the multiperiod model, in order to identify the optimal capacity allocation policies in the dynamic setting.

Unlike the aforementioned publications, this paper investigates bed capacity allocation to balance revenue and equity among multiple demand streams by considering the randomness of both patient arrivals and length of stays from the operational perspective of a large public hospital. Without assuming the probability distributions of the random variables, we develop several data-driven optimization methods to formulate the problem and propose solution approaches to solve it.

The paper is organized as follows: Section 2 describes the practical problem and establishes a single-period newsvendor model. It is then extended in Section 3 to multiperiod models that account for the dynamic nature of the decision process in the real-world scenario. All models shown are verified by numerical examples to illustrate the performance of different data-driven methods in each section. Concluding remarks are made in Section 4.

## 2. Single-Period Model under Certain Capacity

We first describe the practical capacity allocation scenario conducted by WCH's bed schedulers.  Section 2.1 provides the basic newsvendor formulation. In Section 2.2, we present a data-driven method called sample average approximation (SAA) by using past observations of demand.  Section 2.3 extends Section 2.2 by mapping the features associated with demand to obtain the optimal bed allocation quantity.

Due to the self-financing nature of public hospitals in China and their equity responsibility, hospital managers attempt to maximize both revenue and equity when making decisions. Patients backlogged on the waiting queues of each specialty care unit are classified into two categories: urgent elective patients and regular elective patients, which depends on their tolerance thresholds on waiting time and clinical situations. The decision is the number of available beds of the care unit allocated to each category of elective patients each day. Revenue is generated by accepting patients. The revenue a patient generates varies by disease, type of insurance coverage, and length of stay. It is challenging to model real revenue streams in a hospital because the payment mechanisms of the healthcare system are quite complex. Similar to [27], the unit revenue is specific to the patient category. In this setting, we introduce a measure to represent the relative priority of urgent and regular patients, which is called as the adjusted per-unit revenue combining the revenue and equity objective together.

According to the single-period-based decision-making scenario, bed schedulers decide the capacity allocation quota myopically and independently in each period without considering the impact of the decision on continuous periods. Levi et al. [16] proposed that the newsvendor model is one of the most common approaches to solve single-period stochastic optimization problems. In this section, we propose a newsvendor model to balance the trade-off between the allocated bed quota for each patient type and available capacity for them in order to maximize the expected adjusted revenue.

### 2.1. Single-Period Model Formulation

As with known capacity, the uncertainty of the admission control system mainly arises from new arriving demand. Here we formulate a newsvendor model considering two classes of random demand. The common objective is to allocate a bed quantity for each class of demand that maximizes the total expected adjusted revenue with the expression written as

(1) $$\begin{array}{c} ER_{Q_{1}}\left(D_{1},D_{2}\right)=E\left[r_{1}\cdot \min\left\{D_{1},Q_{1}\right\}\right]+E\left[r_{2}\cdot \min\left\{D_{2},\left(K-Q_{1}\right)\right\}\right], \end{array}$$

where *R*_*Q*_1__(*D*_1_, *D*_2_) is the adjusted revenue, *K* is the total fixed bed capacity, *Q*_1_ is the allocated bed quantity for urgent elective demand, and *Q*_2_=*K* − *Q*_1_ represents the remaining quota for regular demand. New arrivals of urgent and regular demand are denoted as *D*_1_ and *D*_2_. The adjusted per-unit revenue gained for serving an urgent demand is *r*_1_ and for a regular demand is *r*_2_.

Given the decision (*Q*_1_, *Q*_2_), the adjusted revenue is then expressed as follows:

(2) $$\begin{array}{c} R_{Q_{1}}\left(x,y\right)=\left\{\begin{array}{cc} r_{1}Q_{1}+r_{2}\left(K-Q_{1}\right), & x\geq Q_{1},\;y\geq K-Q_{1};\\ r_{1}Q_{1}+r_{2}y, & x\geq Q_{1},\;y<K-Q_{1};\\ r_{1}x+r_{2}\left(K-Q_{1}\right), & x<Q_{1},\;y\geq K-Q_{1};\\ r_{1}x+r_{2}y, & x<Q_{1},\;y<K-Q_{1}. \end{array}\right. \end{array}$$

It is acknowledged that the new arrivals of urgent demand and regular demand are independent, so we assume that the probability density function (PDF) of new urgent demand and new regular demand are defined as *f*_*X*_(*x*) and *f*_*Y*_(*y*), respectively. Then, the expected adjusted revenue is expressed as

(3) $$\begin{array}{c} ER_{Q_{1}}\left(x,y\right)=\int_{0}^{Q_{1}}\int_{0}^{K-Q_{1}}\left(r_{1}x+r_{2}y\right)f_{X}\left(x\right)f_{Y}\left(y\right)\text{d}x\text{d}y\\ +\int_{0}^{Q_{1}}\int_{K-Q_{1}}^{\infty }\left(r_{1}x+r_{2}\left(K-Q_{1}\right)\right)f_{X}\left(x\right)f_{Y}\left(y\right)\text{d}x\text{d}y\\ +\int_{Q_{1}}^{\infty }\int_{0}^{K-Q_{1}}\left(r_{1}Q_{1}+r_{2}y\right)f_{X}\left(x\right)f_{Y}\left(y\right)\text{d}x\text{d}y\\ +\int_{Q_{1}}^{\infty }\int_{K-Q_{1}}^{\infty }\left(r_{1}Q_{1}+r_{2}\left(K-Q_{1}\right)\right)f_{X}\left(x\right)f_{Y}\left(y\right)\text{d}x\text{d}y. \end{array}$$

Take the first derivative of *R*_*Q*_1__(*x*, *y*) with respect to *Q*_1_, the optimal decision is given by *Q*_1_^*∗*^ and is expressed as follows:

(4) $$\begin{array}{c} Q_{1}^{*}=\left\{q:r_{1}\int_{q}^{\infty }f_{X}\left(x\right)\text{d}x=r_{2}\int_{K-q}^{\infty }f_{Y}\left(y\right)\text{d}y\right\}. \end{array}$$

Since we use the adjusted per-unit revenues *r*_1_ and *r*_2_ to represent the relative priority of urgent and regular patients, we have *r*_1_ > *r*_2_. According to the optimal *Q*_1_^*∗*^, we can conclude that *P*(*y* ≥ *K* − *Q*_1_) > *P*(*x* ≥ *Q*_1_), indicating that the optimal allocation policy is prone to satisfy urgent demand first.

### 2.2. Observation-Based Data-Driven Newsvendor Problem

When the distributions of urgent demand *D*_1_ and regular demand *D*_2_ are not known, it is one can make use of the historical demand observations to predict *D*_1_ and *D*_2_. Assuming those past *n*-period demand observations are denoted as $\tilde{D_{1}}\left(n\right)=\left\{D_{1,1},D_{1,2},\ldots ,D_{1,n}\right\}$ and $\tilde{D_{2}}\left(n\right)=\left\{D_{2,1},D_{2,2},\ldots ,D_{2,n}\right\}$, then the sample average expected adjusted revenue can be written as

(5) $$\begin{array}{c} \hat{R}\left(Q_{1};\tilde{D_{1}}\left(n\right),\tilde{D_{2}}\left(n\right)\right)=\frac{1}{n}\overset{n}{\underset{i=1}{\sum }}\left[r_{1}\cdot \min\left\{D_{1,i},Q_{1}\right\}+r_{2}\cdot \min\left\{D_{2,i},\left(K-Q_{1}\right)\right\}\right]. \end{array}$$

This approach called SAA is referred to [28], so the optimal decision obtained by the nonparametric method becomes

(6) $$\begin{array}{c} {\hat{Q}}_{1}=\left\{q:r_{1}\int_{q}^{\infty }{\hat{f}}_{X}\left(x\right)\text{d}x=r_{2}\int_{K-q}^{\infty }{\hat{f}}_{Y}\left(y\right)\text{d}y\right\}, \end{array}$$

where ${\hat{f}}_{X}\left(x\right)$ and ${\hat{f}}_{Y}\left(y\right)$ are the empirical probability density functions of the arrival number fitted from the sample with *n* observations. Levi et al. [16] has proved that the objective of the policies obtained by SAA is very close to that of the theoretical optimal policies that are defined with respect to the true underlying demand distributions. The SAA of the newsvendor problem is effective and extremely easy to solve.

### 2.3. Feature-Based Data-Driven Newsvendor Problem

Here, we consider a feature-based data-driven problem. In practice, demand can be affected by various factors, which are observable and available before the allocation decision is made. In the case of WCH, seasonality (day of week, week of month, month of year, etc.), special-day effects (physician vacation, statutory holidays, academic conference, etc.), rehabilitation patients ratio, traffic condition, and weather are new arriving elective demand dependent factors, which are denoted as *x*. In this setting, the newsvendor problem becomes

(7) $$\begin{array}{c} \underset{Q_{1}\left(\cdot \right)\in ℜ,Q_{1}:X⟶\mathbb{R}}{\max}\hat{R}\left(Q_{1}\left(\cdot \right);D\left(x\right)\right)=E\left[\left(r_{1}\cdot \min\left\{D_{1}\left(x\right),Q_{1}\left(x\right)\right\}+r_{2}\cdot \min\left\{D_{2}\left(x\right),K-Q_{1}\left(x\right)\right\}\right)\left|x\right.\right], \end{array}$$

where the decision is now a function that maps the feature space *𝒳* ⊂ *ℝ*^*p*^ to the reals. The expected adjusted revenue function is conditional on the feature vector *x* ∈ *𝒳* ⊂ *ℝ*^*p*^.

**Figure 1:**
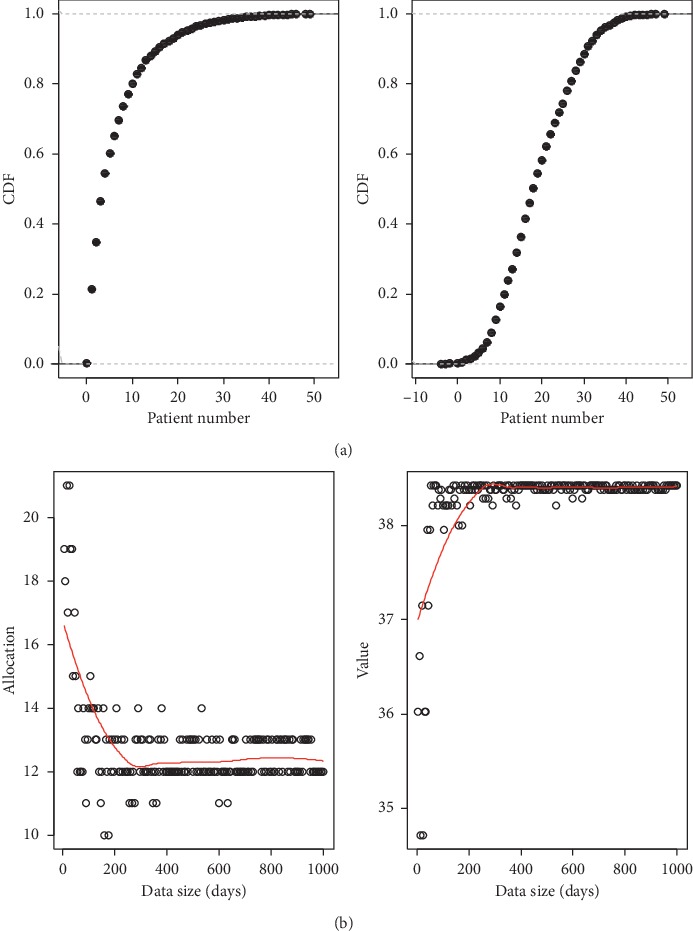
Results of (a) empirical distribution fitting of daily new arrivals of urgent patients and regular patients and (b) optimal capacity allocation policy. In (a), (A) describes the empirical distribution of the daily arrivals of urgent patients, and the (B) describes that of the regular patients. Both charts do not take the related features into account. In (b), (A) describes the optimization results of the allocation quota for urgent patients, and (B) describes the corresponding revenue.

Here, we introduce a nonparametric method that integrates demand feature information into the model. Based on Nadaraya–Watson kernel regression [29, 30], this approach is called the Kernel Optimization (KO) method. For an allocation quota *Q*_1_, the feature-based expected adjusted revenue after observing *x*_*n*+1_ is given by

(8) $$\begin{array}{c} E\left[R\left(Q_{1};D_{1},D_{2}\right)\left|x_{n+1}\right.\right], \end{array}$$

which depends on the demand distributions at *x*_*n*+1_. To estimate *𝔼*[*R*(*Q*_1_; *D*_1_, *D*_2_)|*x*_*n*+1_], we use the Nadaraya–Watson estimator:

(9) $$\begin{array}{c} \frac{\sum_{i=1}^{n}K_{h}\left(x_{n+1}-x_{i}\right)R\left(Q_{1};D_{1,i},D_{2,i}\right)}{\sum_{i=1}^{n}K_{h}\left(x_{n+1}-x_{i}\right)}, \end{array}$$

considering the adjusted revenue to be the dependent variable. *K*_*h*_(·) is a kernel function with bandwidth *h*. Thus, the KO approach focuses on maximizing the above estimator. In this paper, by using the Gaussian kernel density which takes demand seasonality feature into consideration, we simulate the empirical cumulative density functions (CDFs) of urgent demand and regular demand, respectively. Based on the single-period newsvendor model with fixed bed capacity and the optimal allocation policy, we obtain the optimal data-driven allocation quota for urgent elective patients (*Q*_1_) and the optimal objective value (*R*) (Figure 1).

The corresponding CDF of urgent elective patients and that of regular elective patients are shown in Figure 1(a). The results show that the number of urgent patients (with a proportion of 24%) is about one-third of regular patients (with a proportion of 76%), which is typical in WCH. The optimization results of the allocation quota and objective value are presented in Figure 1(b). As can be seen, the bed quantity allocated to urgent patients first decreases with the sample data size, and the optimal objective value tends to increase with the sample data size.

**Remark 1 .**

This is consistent with real practices: when more demand information is available, the senior nurse is then able to match the beds to urgent demand class more accurately and therefore minimize the waste of capacity.

The results here demonstrate the seasonality clearly, with each “season/cycle” lasting for one year. The optimal decision from our model matches the seasonality well and yields improvement over other data-driven models that do not account for external features such as the seasonality. When the running time horizon increases to one year, both the bed quantity allocated to urgent patients and the objective value become asymptotically optimal. According to (4) (particular, in view of *r*_1_ > *r*_2_), the policy is prone to allocate more bed quota to urgent demand in order to satisfy *P*(*x* < *Q*_1_) > *P*(*y* < *K* − *Q*_1_). Hence, we observe that the optimal allocation quota for urgent patients is very large and the optimal value is low when the data size is small. This finding suggests that too much bed capacity is allocated to urgent patients, by which some beds are underutilized and should be allocated to regular patients instead. With more observations included in the data sample, the empirical CDF of urgent patients and regular patients can be simulated more accurately. This approach can identify the final optimal allocation quota for urgent patients and the optimal value obtained from the allocation decision.

In the current practice of WCH, bed schedulers decide the capacity allocation quota myopically and independently in each period without considering the impact of the decision on continuous periods. Considering the dynamic transition of system state, the single-period problem should be extended to a multiperiod problem in the following section, in order to identify the optimal capacity allocation policies in the dynamic setting.

## 3. Multiperiod Model under Random Capacity

In this section, we study the multiperiod extension of the newsvendor problem formulated in Section 2. We consider a single specialty care unit as an illustration, but the models and results can be generalized in other care units directly. In Section 2, we discussed the single-period model with deterministic capacity. With patients on the waiting list or treated in an inpatient ward classified into either “urgent” or “regular,” we consider two multiperiod dynamic models with random bed capacity. In this section, we first present a model with the objective of maximizing the long-run expected adjusted revenue of the admission system and then introduce waiting cost and extra bed cost as the measures of equity into the multiperiod model to examine the specific effects of equity on capacity allocation decision-making.

### 3.1. Multiperiod Model Maximizing Adjusted Revenue

The value function can be written as follows:

(10) $$\begin{array}{c} V_{t}\left(B_{t},X_{t},W_{t}^{1},W_{t}^{2}\right)=\max E\left[r_{1}Q_{t}^{1}+r_{2}Q_{t}^{2}+V_{t+1}\left(B_{t+1},X_{t+1},W_{t+1}^{1},W_{t+1}^{2}\right)\right],\\ Q_{t}^{1}=\min\left\{\alpha \left(X_{t}+\varepsilon_{t}\right),\left(W_{t}^{1}+D_{t}^{1}\right)\right\},\\ Q_{t}^{2}=\min\left\{\left(1-\alpha \right)\left(X_{t}+\varepsilon_{t}\right),\left(W_{t}^{2}+D_{t}^{2}\right)\right\},\\ B_{t+1}={\left[B_{t}-\varepsilon_{t}+Q_{t}^{1}+Q_{t}^{2}\right]}^{+},\\ W_{t+1}^{1}={\left[W_{t}^{1}+D_{t}^{1}-\alpha \left(X_{t}+\varepsilon_{t}\right)\right]}^{+},\\ W_{t+1}^{2}={\left[W_{t}^{2}+D_{t}^{2}-\left(1-\alpha \right)\left(X_{t}+\varepsilon_{t}\right)\right]}^{+},\\ X_{t+1}={\left[X_{t}+\varepsilon_{t}-Q_{t}^{1}-Q_{t}^{2}\right]}^{+}. \end{array}$$

At the beginning of each period *t*, the bed scheduler estimates the value of three random variables: *ε*_*t*_, *D*_*t*_^1^, and *D*_*t*_^2^, denoted as the number of released beds (*ε*_*t*_) and new arriving demand (*D*_*t*_^1^ for urgent patients and *D*_*t*_^2^ for regular patients), respectively. The decision is the ratio *α* according to the state sets (*t*, *B*_*t*_, *X*_*t*_, *W*_*t*_^1^, *W*_*t*_^2^). *W*_*t*_^1^ and *W*_*t*_^2^ represent the number of backlogged urgent patients and regular patients on the waiting list at the beginning of period *t*. The number of unoccupied beds at the beginning of period *t* is denoted as *X*_*t*_, and the number of released beds during period *t* is defined by *ε*_*t*_. The total number of occupied beds in period *t* is represented by *B*_*t*_. *α*(*X*_*t*_+*ε*_*t*_) is the allocated capacity for urgent patients with given information of unoccupied bed capacity *X*_*t*_ while *ε*_*t*_ is stochastic with unknown information. The remaining 1 − *α* ratio of total capacity (*X*_*t*_+*ε*_*t*_) is used to meet the demand of regular patients. Hence, the ultimate fulfilled urgent and regular requests until the end of period *t* are *Q*_*t*_^1^ and *Q*_*t*_^2^. Here the allocation number is the “protection level” that admits patients until the number of patients reaches this level. To solve the data-driven maximization problem, we first characterize the events that occur in each period in this setting as follows:

1. At the beginning of each period *t*, the bed scheduler receives the information of the predictions of released bed capacity (*ε*_*t*_) and new arriving demand (*D*_*t*_^1^ and *D*_*t*_^2^) and then checks patients on the waiting list and the number of unoccupied beds in the inpatient wards, after which (s)he obtains the information of *W*_*t*_^1^, *W*_*t*_^2^, and *X*_*t*_. Based on the above information, the scheduler decides a fraction *α* of (*X*_*t*_+*ε*_*t*_) beds for the urgent patients. If the current waiting census of urgent (regular) patients *W*_*t*_^1^ (*W*_*t*_^2^) is less than the allocated beds *α*(*X*_*t*_+*ε*_*t*_) ((1 − *α*)(*X*_*t*_+*ε*_*t*_)), all urgent (regular) patients on the waiting list are informed of admission in period *t*, and the remaining capacity is used for new arrivals. Otherwise, *α*(*X*_*t*_+*ε*_*t*_) ((1 − *α*)(*X*_*t*_+*ε*_*t*_)) patients on the waiting list are chosen for admission, while other patients including new arrivals need to wait on the waiting list for future available beds.
2. The chosen urgent and regular patients arrive at the hospital and wait for admission.
3. In the morning of the admission day, the information of *B*_*t*_ is recorded through nurse rounds. Afterward rounds, a ratio of inpatients are discharged from hospital and the corresponding beds (*ε*_*t*_) are released. Hence, the total capacity is realized.
4. Throughout the day, emptied beds are assigned to the arriving scheduled patients first, before new arrivals.

The objective of the model is to obtain a dynamic two-class capacity allocation policy that maximizes the expected long-run adjusted revenue of the admission system over a finite horizon. The bed scheduler intends to determine an optimal allocation ratio *α*, in which the setting involves two issues. The first issue is the estimation of three random variables: *ε*_*t*_, *D*_*t*_^1^, and *D*_*t*_^2^, and the second is what features should be considered in order to find the optimal *α*. We adopt the Kernel density to estimate the stochastic variables where the feature is the day of week.

**Figure 2:**
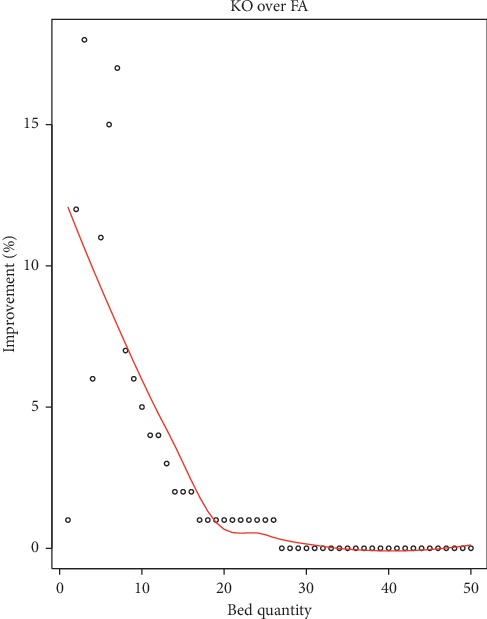
Improvement result of the multiperiod model with random capacity.

Here, we conduct a numerical experiment to verify the dynamic two-class capacity allocation policy. Again as defined in Section 2, we fix *r*_1_=3 and *r*_2_=1. In terms of daily patient arrivals, the urgent segment follows *D*_*t*_^1^=int(|sin(*t*/*τ*^1^)| · *μ*^1^+*σ*^1^ · *N*(1, 0.1^2^)) and the regular segment follows *D*_*t*_^2^=int(|sin(*t*/*τ*^2^)| · *μ*^2^+*σ*^2^ · *N*(1, 0.1^2^)), where *τ*^1^(*τ*^2^) and *σ*^1^(*σ*^2^) represent the cycle and variance of the urgent (regular) segment, respectively. Here we fix *τ*^1^=*τ*^2^=3 and *σ*^1^=*σ*^2^=1. Similarly, we assume the daily released bed capacity is a cyclical function and it follows *ε*_*t*_=int((|sin(*t*/6)| · *N*(0.5, 0.2^2^))*B*_*t*_). The sample data, comprised of 50 periods and controlled with an initial allocation ratio *α*=0.6, are randomly generated. To compare the reward under each control method, we evaluate each method on 500 randomly generated problem instances, and the horizon of each instance is *T*=50. We vary the total capacity from 1 to 50. The improvement of kernel optimization (KO) over fixed ratio allocation (FA) (in which the hospital estimates the daily released beds with the mean value, i.e., *τ*=(1/*T*)∑_*t*=1_^*T*^*ε*_*t*_/*B*_*t*_) is presented in Figure 2.

Results from the simulation show that the improvement is remarkable when the total capacity is medium, while it decreases with the number of total bed quantity. When the total bed capacity increases from 1 to 20, the improvement of KO over FA decreases from 12% to 1%. When the total capacity is over 20, there is almost no improvement for using KO over FA. Confronted with insufficient bed capacity, resource preemption between urgent and regular demand is common, and it is more challenging for the hospital bed manager to allocate the capacity quota as closely as possible to both urgent and regular demand so that the opportunity costs resulted from shortage and overage of bed capacity can be minimized. In this setting, FA adopts the fixed quota which does not consider the dynamic variations of the system. This also verifies that KO offers a great value in dynamic capacity allocation when the total bed capacity is insufficient.

### 3.2. Multiperiod Model Maximizing Both Revenue and Equity

Introducing waiting cost and extra bed cost as the measurements of equity into the multiperiod model, the objective function becomes

(11) $$\begin{array}{c} R=\max\frac{1}{T}\overset{T}{\underset{t=1}{\sum }}\left(R_{t}-O_{t}-P_{t}\right), \end{array}$$

where *R*_*t*_ denotes the revenue on a given day *t*. *O*_*t*_ is the waiting cost, and *P*_*t*_ denotes the extra bed cost generated. *R*_*t*_ is expressed as

(12) $$\begin{array}{c} R_{t}=\overset{Q_{t}}{\underset{i=1}{\sum }}U_{i}\left(x_{i,t}\right)=\overset{Q_{t}^{1}}{\underset{i=1}{\sum }}U_{i}^{1}\left(x_{i,t}\right)+\overset{Q_{t}^{2}}{\underset{j=1}{\sum }}U_{j}^{2}\left(x_{j,t}\right)=r_{1}Q_{t}^{1}+r_{2}Q_{t}^{2}, \end{array}$$

where *U*_*i*_(*x*_*i*,*t*_) represents the utility of patient *i* in period *t* given feature *x*_*i*,*t*_.

The data-driven elective admission control problem is given by

(13) $$\begin{array}{c} f=R=\max\frac{1}{T}\overset{T}{\underset{t=1}{\sum }}\left[r_{1}Q_{t}^{1}+r_{2}Q_{t}^{2}-O_{t}-P_{t}\right], \end{array}$$

where

(14) $$\begin{array}{c} Q_{t}^{1}+Q_{t}^{2}\leq K_{t},\\ K_{t}^{0}=K_{t}-Q_{t}^{1}-Q_{t}^{2}=\xi_{t}K_{t},\\ W_{t+1}^{1}=W_{t}^{1}-Q_{t}^{1}+D_{t}^{1},W_{t+1}^{2}=W_{t}^{2}-Q_{t}^{2}+D_{t}^{2},\\ B_{t+1}^{1}=B_{t}^{1}\left(1-\gamma_{t}^{1}\right)+Q_{t}^{1}+E_{t},B_{t+1}^{2}=B_{t}^{2}\left(1-\gamma_{t}^{2}\right)+Q_{t}^{2},\\ X_{t+1}=X_{t}+B_{t}^{1}\gamma_{t}^{1}+B_{t}^{2}\gamma_{t}^{2}-E_{t}-Q_{t}^{1}-Q_{t}^{2},\\ B_{t}^{1}+B_{t}^{2}+X_{t}=K,\\ Q_{t}^{1}=\left(1-\xi_{t}\right)K_{t}\left[\frac{\alpha W_{t}^{1}}{\left(W_{t}^{1}+W_{t}^{2}\right)}\right]\left[\overset{5}{\underset{k=1}{\prod }}\left(1-\beta_{k}d_{k,t}\right)\right],\\ O_{t}=\rho_{1}\left(W_{t}^{1}-Q_{t}^{1}\right)+\rho_{2}\left(W_{t}^{2}-Q_{t}^{2}\right),\\ P_{t}=b{\left[\left(E_{t}+Q_{t}^{1}+Q_{t}^{2}\right)-\left(X_{t}+B_{t}^{1}\gamma_{t}^{1}+B_{t}^{2}\gamma_{t}^{2}\right)\right]}^{+}, \end{array}$$

where 0 ≤ *ξ*_*t*_, *γ*_*t*_^1^, *γ*_*t*_^2^ ≤ 1, *α* ≥ 0, *β*_*k*_ ≥ 0, ∀*t* ∈ [1, *T*], ∀*k* ∈ [1,5]. *d*_*k*,*t*_ is defined as follows:

(15) $$\begin{array}{c} d_{k,t}=\left\{\begin{array}{cc} 1, & \text{if day }t\text{ is the }k\text{th weekday};\\ 0, & \text{otherwise,} \end{array}\right. \end{array}$$

where *K*_*t*_ is a given prediction of available capacity for day *t*. *K*_*t*_^0^ is the reservation quantity of bed capacity for day *t* and accounts for a proportion *ξ*_*t*_ of *K*_*t*_. *X*_*t*_ represents the amount of extra bed when *X*_*t*_ is negative or an unoccupied bed when *X*_*t*_ is positive at the beginning of day *t*. The total available capacity on each day is the sum of *X*_*t*_ and the amount of discharged patients. We denote *B*_*t*_^1/2^ as the urgent and regular inpatient census at the beginning of day *t* and *γ*_*t*_^1/2^ as the discharge percentage of each class of inpatients. The actual admitted census originates from the sum of *Q*_*t*_=*Q*_*t*_^1^+*Q*_*t*_^2^ and *E*_*t*_, where *E*_*t*_ represents those who are admitted the day they submit their requests. *K* represents the total number of standard beds belonging to the care unit, which is a constant. *W*_*t*_^1^/(*W*_*t*_^1^+*W*_*t*_^2^) represents the composition structure of the waiting list, and *α* is the coefficient with respect to the feature. ∏_*k*=1_^5^(1 − *β*_*k*_*d*_*k*,*t*_) involves the day of the week feature, in which *β*_*k*_ represents the reservation ratio of capacity in that weekday. The larger the value of *α*, the more the capacity will be allocated to urgent patients, while the larger the value of *β*_*k*_, the less the capacity will be allocated to urgent patients. Suppose each urgent patient incurs a waiting cost of *ρ*_1_ per day, and *ρ*_2_ per day for each regular patient, we assume each extra bed incurs a penalty cost *b*.

#### 3.2.1. Data

Data of our study were collected from the Admission Management Information System, a subsystem of the Hospital Information System (HIS) of WCH. Raw data for the urology specialty unit cover the period of January to October 2015 (only workdays recorded) and include three sets: (1) all admission observations (3507), (2) all cancelation records (540), and (3) all requests that are still waiting for beds (260). The waiting list data as the input to the multiperiod models are generated by integrating these three sets of raw data recorded daily at the patient level as shown in Table 1.

Table 2 presents some information about the current practice of WCH. As can be seen, the average waiting time of elective admissions at WCH is 49 days, and the maximum value is 332 days implying a heavily skewed property. The number of elective patients on the waiting list presents an increasing trend, which interprets the main reason of long waiting time. The evidence of random bed capacity can be captured by the number of daily discharged patients [31]. The data show that the historical admission quotas decided by schedulers often exceed the number of released bed capacity, which may result in delays of emergency admissions and congestion of emergency department. Moreover, the total number of standard beds belonging to the urology specialty care unit is 140, and the average number of inpatients in the wards is around 140, which results in a nearly 100% bed utilization. The available bed capacity is particularly insufficient for serving all waiting list demand and new arriving demand. In this setting, extra beds are always used, and putting patients on extra beds in corridors could generate additional staff costs and simultaneously worsens a patient's care quality and care delivery environment. The data show that 66.85% of the days involved using extra beds. Moreover, the number of daily admitted urgent patients takes a proportion of around 30%. We could also identify the impact of day of the week feature in daily total elective admissions and realized urgent cases is significant, indicating that schedulers take into account the day of the week effect of capacity and demand when deciding the admission quotas.

As a large teaching and research hospital, it would be beneficial for WCH managers to consider the seasonality pattern of demand and capacity, teaching cycle, and academic symposium into the decision-making of bed capacity allocation problem. Hence, it is essential to introduce feature-based methods to the newsvendor framework, in order to evaluate the value of capturing information on the random capacity and demand. According to the model formulated in equation (13), the objective function (*f*) is comprised of the revenue (*r*_1_*Q*_*t*_^1^+*r*_2_*Q*_*t*_^2^) generated by accepting admission patients, the waiting cost (*ρ*_1_(*W*_*t*_^1^ − *Q*_*t*_^1^)+*ρ*_2_(*W*_*t*_^2^ − *Q*_*t*_^2^)), and the extra bed cost (*b*[(*E*_*t*_+*Q*_*t*_^1^+*Q*_*t*_^2^) − (*X*_*t*_+*B*_*t*_^1^*γ*_*t*_^1^+*B*_*t*_^2^*γ*_*t*_^2^)]^+^) by measuring the penalty of equity. One control variable of *f* is *ξ* since the decision of *ξ*_*t*_ directly determines the sum of *Q*_*t*_^1^ and *Q*_*t*_^2^. In addition, *f* is also sensitive to the revenue rate and cost rate parameters (*r*_1_, *r*_2_, *ρ*_1_, *ρ*_2_, and *b*), which makes these parameters significant in optimal search of *f*, *ξ*, *Q*_*t*_^1^, and *Q*_*t*_^2^. It is acknowledged that we consider two features in terms of finding the optimal allocation quantity: (1) the day of the week and (2) the composition structure of the waiting list. Hence, the control variables of *Q*_*t*_^1^ are *α* and *β*_*k*_.

We apply the data set to a Matlab program to generate computational results of the model. We first conducted sensitivity analyses on control variables (*ξ*, *α* and *β*_*k*_) with fixed values of revenue rate and cost rate parameters (*r*_1_, *r*_2_, *ρ*_1_, *ρ*_2_, and *b*). Subsequently, sensitivity analyses on *r*_1_, *r*_2_, *ρ*_1_, *ρ*_2_, and *b* were conducted to compare the optimal results with that obtained from a fixed setting of these parameters.

#### 3.2.2. Sensitivity Analysis on Control Variables

**Figure 3:**
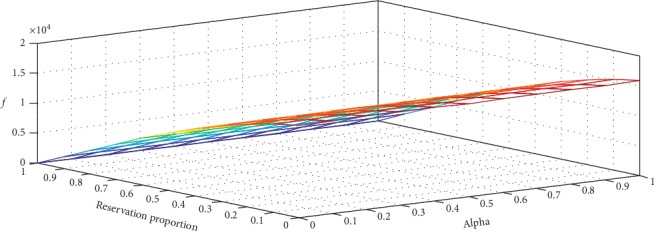
The optimal objective value *f*(*ξ*, *α*) changing with the capacity reservation proportion and the allocation quota for urgent patients.

We assume that the unit adjusted revenue of an urgent patient is 1.25 times as that of a regular patient, with each extra bed case incurring a penalty cost. We use *T*=193 past observations of *K*_*t*_, *W*_*t*_^1/2^, *B*_*t*_^1/2^, *γ*_*t*_^1/2^, and *E*_*t*_ to compute the optimal value of *f*, *Q*_*t*_^1/2^, *ξ*_*t*_, *α*, and *β*_*k*_. The results are shown in Table 3 and Figure 3.

The results show that *f* declines with *ξ* strictly while without strictly monotonic property on *α*. The maximum value of *f* is marked with a double asterisk ^*∗∗*^ in the table with *α*=1, *β*=(0,0.6, 0.6, 0.1, 0.9) and *ξ*=0. *ξ*=0 indicates that it is optimal to exhaust all expected capacity without reservation for the next period. *α*=1 implies that the available capacity should be assigned to urgent patients as much as possible. The findings also show that capacity quantity allocated to urgent patients declines with the value of *β*_*k*_.

It is interesting to note from the *β*_*k*_ values that more beds should be allocated to urgent patients on Monday and Thursday than the other weekdays. In contrast, in the current practice of WCH, more urgent patients are admitted on Tuesday and Wednesday, and, hence, our result provides a useful insight for improving the current practice. Since surgeries are performed during weekdays, elective patients are also admitted during weekdays, and patient discharge rate on Friday is high [31], resulting in a relatively low bed occupancy rate during weekends. The remaining inpatients are those postoperative patients who require rehabilitation treatment. Our results suggest that admitting more urgent patients on Monday can be beneficial for physician's surgery scheduling in the new week. Moreover, the average length of stay of patients treated in the urology specialty care unit is around 10 days, which forms a cycle for physicians to admit more urgent patients on Monday and Thursday, in order to supplement more surgeries for the high discharge rate on Friday.

**Figure 4:**
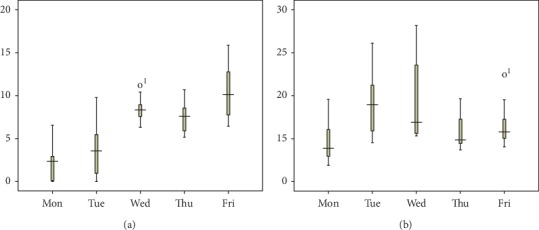
A boxplot of the number of (a) extra beds and (b) holding beds by the day of the week pattern.

**Figure 5:**
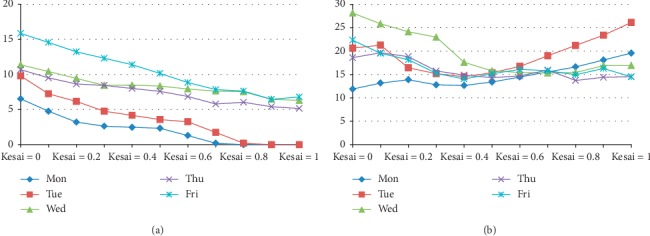
A plot of the number of (a) extra beds and (b) holding beds varying with *ξ* by the day of the week pattern.

**Figure 6:**
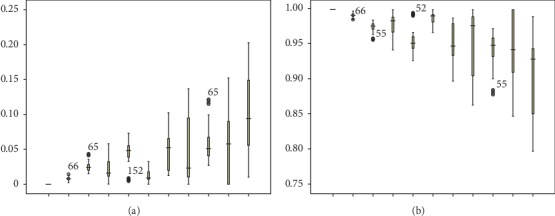
A boxplot of the proportion of (a) *Q*_1_ and (b) *Q*_2_ varying with *ξ*.

Since the quantity of extra bed and that of unoccupied bed affect the total revenue directly, we further provide the day of the week pattern of these two quantities in Figure 4. It is obvious that the degree of bed shortage worsens from Monday to Friday, while the number of bed overage first increases from Monday to Tuesday before it declines. The number of extra beds presented in Figure 5(a) declines with the value of *ξ* on each day of the week, implying that the more capacity reserved for the next period, the fewer the number of extra beds. We can conclude that the number of extra beds is a decreasing function of reservation proportion. Moreover, the number of extra beds strictly decreases from Friday to Wednesday, Thursday, Tuesday, and Monday, regardless of the value of the reservation proportion. Results from Figure 5(b) show that the number of unoccupied beds on Monday and Tuesday first increases with the value of *ξ*, then declines, and finally increases with that of *ξ*. For Wednesday, Thursday, and Friday, the unoccupied bed volume shows a downward trend when the value of *ξ* varies from 0 to 1. We also find that the unoccupied bed quantity can gain balance during the week when *ξ*=0.5. As concluded above, the objective function *f* is a strictly decreasing function of *ξ*, we expound on how urgent admissions volume (*Q*_1_) and regular admissions volume (*Q*_2_) change with the value of reservation proportion (*ξ*), and the results are shown in Figure 6. The admission quota of urgent patients generally increases with the value of *ξ*, suggesting more urgent patients should be admitted when available capacity in this period is tight.

#### 3.2.3. Sensitivity Analysis on Revenue and Cost Parameters

**Figure 7:**
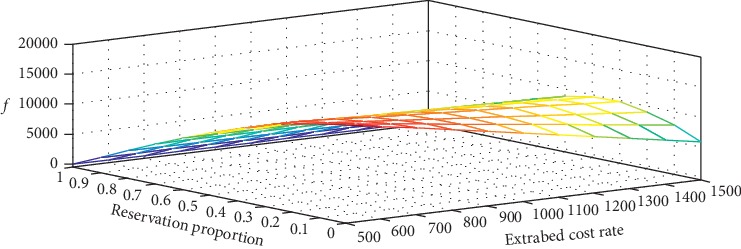
The optimal objective value *f*(*ξ*, *b*) changing with the capacity reservation proportion and the extra bed cost rate.

With a similar setting in Section 3.2.2, i.e., the unit adjusted revenue of an urgent patient being 1.25 times as that of a regular patient, we change the value of extra bed cost rate *b* to capture the trend of the optimal *f*(*α*, *β*_*k*_, *ξ*). The results are shown in Table 4 and Figure 7. In Table 4, the maximum value of *f* is marked with an asterisk ^*∗*^ when the value of *b* is changed. We find that *f* is decreasing in *ξ* when *b* ≤ 700, while first increases with *ξ* then declines when *b* > 700. The results suggest that it is beneficial to exhaust all available capacity in each period without reservation to the next period when the penalty for using extra beds is small. When the extra bed penalty cost reaches a certain level, it is better to reserve a specific quota of available capacity in each period to the next period. From the results, it is also clear that the optimal reservation quota *ξ* is nondecreasing in extra bed cost rate *b* and the optimal *f* is decreasing in *b*. With the increase of *b*, the maximum ratio of available capacity that can be reserved to the next period is 0.3.

In WCH, the extra bed cost rate can be affected by the numbers of available extra beds and the patients placed on extra beds. On one hand, since all extra beds are viewed as a resource pool and are shared by 44 specialty care units, when the number of available extra beds is tight, using one extra bed for a patient may lead to a higher cost for transferring a more severe patient to other hospitals. On the other hand, the staffing level of physicians and nurses are roughly fixed during a schedule shift, more patients on extra beds would add excessive workload to clinical staffs, which simultaneously leads to extra wage costs. Therefore, the extra bed cost rate *b* defined in our model reflects the practical availability of extra beds and the workload of clinical staffs, i.e., the lower the *b*, the higher the availability and the lower the workload. In the current practice of WCH, the decision-maker always exhausts all beds to avoid possible idle cost without considering the cost rate of extra beds. The results on sensitivity analysis in this paper suggest that it is beneficial to exhaust all beds with a higher availability of extra beds; otherwise, reserving a certain proportion of total bed capacity available in this period for use in the next period may improve profitability.

## 4. Conclusions

Public hospital managers are under great pressure to pursue the trade-off between revenue and equity when allocating limited and critical bed capacity. Inpatient beds can serve as a key revenue source for hospitals but also consume expensive resources when not managed effectively. In the presence of capacity and demand variability, hospital managers must balance the trade-off between being overloaded or underloaded to mitigate the risk of long waiting time for urgent patients, high extra bed quantity, or excess idling (holding bed).

In this study, we first proposed several data-driven methods under different conditions for solving the bed capacity allocation problem in a Chinese public hospital. By using simulation methods and hospital historical data, we demonstrated how heterogeneity in demand types, choices of analytical models, and data availability can affect the performance of healthcare operational decision. First, formulating the real-world decision scenario, we provided a feature-based single-period model that takes demand seasonality feature into consideration to optimize the single-period static allocation decision and the objective function. Considering the dynamic transition of system state, we then extended this single-period static decision problem to a multiperiod dynamic decision problem, in order to identify the optimal capacity allocation policies in the dynamic setting. We formulated two multiperiod models with random capacity. The difference between the two models is that we only consider adjusted revenue in the first model, while inherent waiting cost and extra bed cost are introduced as measurements of equity in the second model. Through numerical experiments by using real data from a large public hospital in Chengdu, China, the final optimal allocation quota for each patient category and the optimal value obtained from the allocation decision of the single-period model have been characterized. In terms of the multiperiod models, we first verified that the kernel optimization method performs better than the fixed quota allocation policy with significant improvement when the total bed capacity is medium. It is intuitive that the performance of improvement will decrease with the number of available bed quantity. For the second dynamic model, we showed that the day of the week feature and the composition structure of waiting urgent patients and elective patients have remarkable impact on the optimal allocation decision and the objective value. Our findings offer some important insights to hospital managers, i.e., the optimal revenue for hospital will decrease when there are more urgent elective patients on the waiting list. It is optimal to allocate more capacity to urgent patients on Monday and Thursday than other weekdays as the capacity allocated to urgent patients declines with the coefficients of the day of the week effect.

This paper contributes to the literature by (1) considering the joint optimization of revenue and equity in healthcare capacity allocation problems; (2) proposing three data-driven optimization methods varying from single-period static model to multiperiod dynamic model; and (3) characterizing the performance of distinct optimization methods using real data from the collaborative hospital. However, several limitations also exist in this work. It is challenging to model real revenue streams in a hospital because the payment mechanisms of the healthcare system are quite complex. Revenues are averaged across all diseases, insurance coverage types, and length of stays within each patient category. Moreover, indexes of equity of access and responsiveness in previous works are defined from various regional and hospital operational views. This study integrated the two objectives of revenue and equity into a measure called as the unit adjusted revenue. Capacity allocation among multiple patient categories is a complex issue, particularly modeling the revenue and equity without these assumptions. Future research can be pursued in several directions. One is to create measures that can model real revenue and equity streams, which can benefit the identification of effects of each stream on the optimization objective. Another extension is to characterize the analytical structure properties of the multiperiod dynamic models, and then comparisons between theoretical optimal policies and data-driven optimal policies can be conducted to verify the effectiveness of algorithms. The third is the joint scheduling of multiple resources such as beds, operating rooms, surgeons, and nurses.

In summary, we have developed a data-driven stochastic optimization framework to explore how information, data, and estimation methods can be used to support the bed capacity allocation decision. The methods could be generalized to other healthcare stochastic optimization settings involving demand with predictable heterogeneity in future.

## Figures and Tables

**Table 1:** The format of patient information collection on waiting list.

Patient ID	Gender	Admission certificate date	Arrival date	Admission date	Diagnosis	Disease type	Insurance type	Waiting time	Dual referral

**Table 2:** Summary statistics: average and percentage.

-	Monday (39 days)	Tuesday (39 days)	Wednesday (38 days)	Thursday (39 days)	Friday (38 days)
Total inpatient census when choosing	132	138	141	140	150
Total waiting census when choosing	332	332	350	348	341
Waiting time (days)	50	49	49	48	50
Male	68.10%	69.54%	67.87%	67.87%	67.83%
Urgent	16.57%	14.03%	16.67%	16.27%	16.54%
Daily total admissions	27	13	11	19	15
Daily chosen admissions	21 (78.42%)	8 (65.03%)	8 (74.31%)	14 (76.62%)	11 (76.27%)
Daily chosen urgent admissions	23.68%	28.10%	34.89%	26.57%	22.35%
Waiting time of daily admissions	21	12	11	16	18

**Table 3:** Computational results.

	*α*=0	*α*=0.1	*α*=0.2	*α*=0.3	*α*=0.4	*α*=0.5	*α*=0.6	*α*=0.7	*α*=0.8	*α*=0.9	*α*=1
*β*_1_	0.5	0.3	0.2	0.7	0.1	0.8	0.4	0.9	0.6	1	0^*∗*^
*β*_2_	0	0.8	0.3	0.7	0.9	1	0.4	0.1	0.5	0.2	0.6^*∗*^
*β*_3_	0	0.5	0.4	0.1	0.3	0.9	0.2	0.8	0.7	1	0.6^*∗*^
*β*_4_	0	0.5	0.3	0.4	0.2	0.9	0.8	1	0.7	0.6	0.1^*∗*^
*β*_5_	0.6	0.5	0	1	0.2	0.7	0.8	0.1	0.3	0.4	0.9^*∗*^
*f*(*ξ*=0)	15482	15521	15608	15589	15701	15539	15706	15719	15771	15770	15952^*∗∗*^
*f*(*ξ*=0.1)	14764	14799	14877	14860	14961	14815	14965	14977	15023	15023	15187^*∗*^
*f*(*ξ*=0.2)	13826	13858	13927	13912	14001	13871	14005	14016	14057	14056	14202^*∗*^
*f*(*ξ*=0.3)	12628	12656	12716	12703	12782	12668	12785	12794	12830	12830	12957^*∗*^
*f*(*ξ*=0.4)	11227	11251	11303	11292	11359	11261	11361	11369	11400	11400	11509^*∗*^
*f*(*ξ*=0.5)	9603.9	9623.4	9666.7	9657.4	9713.3	9632	9715.5	9722.4	9748	9747.7	9838.8^*∗*^
*f*(*ξ*=0.6)	7827.7	7843.3	7877.9	7870.5	7915.2	7850.1	7917	7922.5	7943	7942.7	8015.6^*∗*^
*f*(*ξ*=0.7)	5934.9	5946.6	5972.5	5967	6000.5	5951.7	6001.8	6006	6021.4	6021.1	6075.8^*∗*^
*f*(*ξ*=0.8)	3974.7	3982.5	3999.8	3996.1	4018.5	3986	4019.4	4022.1	4032.4	4032.2	4068.7^*∗*^
*f*(*ξ*=0.9)	1974.4	1978.3	1987	1985.1	1996.3	1980	1996.7	1998.1	2003.2	2003.2	2021.4^*∗*^
*f*(*ξ*=1)	−36.269	−36.269	−36.269	−36.269	−36.269	−36.269	−36.269	−36.269	−36.269	−36.269	−36.269

**Table 4:** Computational results.

	*b*=500	*b*=600	*b*=700	*b*=800	*b*=900	*b*=1000	*b*=1100	*b*=1200	*b*=1300	*b*=1400	*b*=1500
*f*(*ξ*=0)	15952^*∗*^	14941^*∗*^	13931^*∗*^	12920	11909	10898	9887	8876.1	7865.2	6854.3	5843.5
*f*(*ξ*=0.1)	15187	14443	13699	12955^*∗*^	12211^*∗*^	11468	10724	9980	9236.2	8492.3	7748.5
*f*(*ξ*=0.2)	14202	13682	13161	12640	12120	11599^*∗*^	11079	10558	10037	9516.6	8996
*f*(*ξ*=0.3)	12957	12937	12587	12238	11888	11539	11189^*∗*^	10840^*∗*^	10490^*∗*^	10141^*∗*^	9791.4^*∗*^
*f*(*ξ*=0.4)	11509	11572	11353	11134	10915	10696	10477	10258	10039	9820.4	9601.5
*f*(*ξ*=0.5)	9838.8	9940.9	9808	9675.1	9542.2	9409.3	9276.4	9143.5	9010.6	8877.7	8744.8
*f*(*ξ*=0.6)	8015.6	8126.2	8048.8	7971.4	7893.9	7816.5	7739.1	7661.7	7584.3	7506.9	7429.5
*f*(*ξ*=0.7)	6075.8	6171.6	6126.3	6081.1	6035.9	5990.6	5945.4	5900.2	5854.9	5809.7	5764.5
*f*(*ξ*=0.8)	4068.7	4042.2	4015.6	3989.1	3962.6	3936.1	3909.5	3883	3856.5	3829.9	3803.4
*f*(*ξ*=0.9)	2021.4	2005.5	1989.7	1973.8	1958	1942.1	1926.3	1910.4	1894.6	1878.7	1862.8
*f*(*ξ*=1)	−36.269	−43.523	−50.777	−58.031	−65.285	−72.539	−79.793	−87.047	−94.301	−101.55	−108.81

## Data Availability

The data used to support the findings of this manuscript are restricted by the West China Hospital in order to protect patient privacy and avoid legal and ethical risks. Data are available from the West China Hospital for researchers who meet the criteria for access to confidential data.

## References

[1] Zhou L., Geng N., Jiang Z., Wang X. (2018). Multi-objective capacity allocation of hospital wards combining revenue and equity. *Omega*.

[2] Helm J. E., Van Oyen M. P. (2014). Design and optimization methods for elective hospital admissions. *Operations Research*.

[3] Culyer A. J., Wagstaff A. (1993). Equity and equality in health and health care. *Journal of Health Economics*.

[4] Wagstaff A., van Doorslaer E. (2000). Chapter 34 equity in health care finance and delivery. *Handbook of Health Economics*.

[5] Starfield B. (2009). Primary care and equity in health: the importance to effectiveness and equity of responsiveness to peoples’ needs. *Humanity & Society*.

[6] Xiao Q., Luo L., Zhao S.-Z., Ran X.-B., Feng Y.-B. (2018). Online appointment scheduling for a nuclear medicine department in a Chinese hospital. *Computational and Mathematical Methods in Medicine*.

[7] Liu S., Griffiths S. M. (2011). From economic development to public health improvement: China faces equity challenges. *Public Health*.

[8] Zhang L., Li M., Ye F., Ding T., Kang P. (2016). An investigation report on Large Public Hospital Reforms in China.

[9] Reynolds L., Mckee M. (2011). Serve the people or close the sale? Profit-driven overuse of injections and infusions in China’s market-based healthcare system. *The International Journal of Health Planning and Management*.

[10] Zipkin P. (2000). *Foundations of Inventory Management*.

[11] Ben-Tal A., Nemirovski A. (1998). Robust convex optimization. *Mathematics of Operations Research*.

[12] Bertsimas D., Sim M. (2004). The price of robustness. *Operations Research*.

[13] Liyanage L. H., Shanthikumar J. G. (2005). A practical inventory control policy using operational statistics. *Operations Research Letters*.

[14] Chen D., Qi J., Meng F., Ang J., Chu S., Sim M. (2015). A robust optimization model for managing elective admission in hospital. *Operations Research*.

[15] Huh W. T., Levi R., Rusmevichientong P., Orlin J. B. (2011). Adaptive data-driven inventory control with censored demand based on kaplan-meier estimator. *Operations Research*.

[16] Levi R., Roundy R. O., Shmoys D. B. (2007). Provably near-optimal sampling-based policies for stochastic inventory control models. *Mathematics of Operations Research*.

[17] Levi R., Perakis G., Uichanco J. (2015). The data-driven newsvendor problem: new bounds and insights. *Operations Research*.

[18] Ban G.-Y., Rudin C. (2015). The big data newsvendor: practical insights from machine learning. *Operations Research*.

[19] Cox I. J., Ghosn J., Yianilos P. N. Feature-based face recognition using mixture-distance.

[20] Calhoun V. D., Adali T. (2009). Feature-based fusion of medical imaging data. *IEEE Transactions on Information Technology in Biomedicine*.

[21] Tsitsiklis J. N., Van Roy B. (1996). Feature-based methods for large scale dynamic programming. *Machine Learning*.

[22] Barz C., Rajaram K. (2016). Elective patient admission and scheduling under multiple resource constraints. *Production and Operations Management*.

[23] Young J. P. (1963). *A Queuing Theory Approach to the Control of Hospital Inpatient Census*.

[24] Gerchak Y., Gupta D., Henig M. (1996). Reservation planning for elective surgery under uncertain demand for emergency surgery. *Management Science*.

[25] Ayvaz N., Huh W. T. (2010). Allocation of hospital capacity to multiple types of patients. *Journal of Revenue and Pricing Management*.

[26] Huh W. T., Liu N., Truong V.-A. (2013). Multiresource allocation scheduling in dynamic environments. *Manufacturing and Service Operations Management*.

[27] Pinker E., Tezcan T. (2013). Determining the optimal configuration of hospital inpatient rooms in the presence of isolation patients. *Operations Research*.

[28] Shapiro A., Dentcheva D., Ruszczynski A. (2009). *Lectures on Stochastic Programming: Modeling and Theory*.

[29] Nadaraya E. A. (1964). On estimating regression. *Theory of Probability & Its Applications*.

[30] Watson G. S. (1964). Smooth regression analysis, Sankhyā. *The Indian Journal of Statistics, Series A*.

[31] Zhu T., Luo L., Zhang X., Shi Y., Shen W. (2017). Time-series approaches for forecasting the number of hospital daily discharged inpatients. *IEEE Journal of Biomedical and Health Informatics*.

